# Influence of Fractionation Methods on Physical and Biological Properties of Injectable Platelet-Rich Fibrin: An Exploratory Study

**DOI:** 10.3390/ijms20071657

**Published:** 2019-04-03

**Authors:** Prakan Thanasrisuebwong, Rudee Surarit, Sompop Bencharit, Nisarat Ruangsawasdi

**Affiliations:** 1 Department of Oral and Maxillofacial Surgery, Faculty of Dentistry, Mahidol University, Bangkok 10400, Thailand; ae13ea@gmail.com; 2 Ph.D. program in Oral Biology, Faculty of Dentistry, Mahidol University, Bangkok 10400, Thailand; 3 Department of Oral Biology, Faculty of Dentistry, Mahidol University, Bangkok 10400, Thailand; rudee.sur@mahidol.ac.th; 4 Department of General Dentistry, School of Dentistry, Virginia Commonwealth University, Richmond, VA 23298, USA; sbencharit@vcu.edu; 5 Department of Biomedical Engineering, College of Engineering, Virginia Commonwealth University, Richmond, VA 23298, USA; 6 Department of Pharmacology, Faculty of Dentistry, Mahidol University, Bangkok 10400, Thailand

**Keywords:** bone regeneration, fractionation, growth factors, i-PRF, PRF, ROTEM^®^

## Abstract

Injectable platelet-rich fibrin (i-PRF) has been used as an autografting material to enhance bone regeneration through intrinsic growth factors. However, fractionation protocols used to prepare i-PRF can be varied and the effects of different fractionation protocols are not known. In this study, we investigated the influence of different fractions of i-PRF on the physical and biological properties derived from variations in i-PRF fractionation preparation. The i-PRF samples, obtained from the blood samples of 10 donors, were used to harvest i-PRF and were fractioned into two types. The yellow i-PRF fractionation was harvested from the upper yellow zone, while the red i-PRF fractionation was collected from both the yellow and red zone of the buffy coat. The viscoelastic property measurements, including the clot formation time, α-angle, and maximum clot firmness, were performed by rotational thromboelastometry. The fibrin network was examined using a scanning electron microscope. Furthermore, the concentration of growth factors released, including VEGF, TGF-β1, and PDGF, were quantified using ELISA. A paired *t*-test with a 95% confidence interval was used. All three viscoelastic properties were statistically significantly higher in the yellow i-PRF compared to the red i-PRF. The scanning electron microscope reviewed more cellular components in the red i-PRF compared to the yellow i-PRF. In addition, the fibrin network of the yellow i-PRF showed a higher density than that in the red i-PRF. There was no statistically significant difference between the concentration of VEGF and TGF-β1. However, at Day 7 and Day 14 PDGF concentrations were statistically significantly higher in the red i-PRF compared to the yellow group. In conclusion, these results showed that the red i-PRF provided better biological properties through the release of growth factors. On the other hand, the yellow i-PRF had greater viscoelastic physical properties. Further investigations into the appropriate i-PRF fractionation for certain surgical procedures are therefore necessary to clarify the suitability for each fraction for different types of regenerative therapy.

## 1. Introduction

Platelet-rich fibrin (PRF) derived from the centrifugation of whole blood has long been used successfully in regenerative dentistry and medicine [1,2,3,4,5]. A centrifugation speed of 2700 rpm for 12 min in PRF with a glass or glass-coated tubes permits the use of PRF material without any additional anticoagulants. The fibrin matrix scaffold network in PRF appears to be beneficial in tissue regeneration [6]. PRF has shown to enhance regeneration of bone [1,7], cartilage [8], soft tissue [5,9,10,11], and nerve fiber [12]. However, the application of solid PRF, resulting from a blood sample preparation at 400 g relative centrifugal force (RCF) or 2700 round per minute (rpm) for 12 min, has its limitations. For instance, it cannot be mixed with a particulate bone substitute. To mix with particulate bone, the biomaterials in a liquid form are more suitable. Both injectable platelet-rich fibrin (i-PRF) and PRF-predecessor platelet-rich plasma (PRP) are the liquid formula within the platelet concentrated group that can be used efficiently for mixing with the particulate bone graft. PRP is traditionally prepared from whole blood using a higher speed centrifugation compared to PRF [1,2,3,4,5]. Unfortunately, PRP has some drawbacks because it requires either synthetic anticoagulants, such as citrate dextrose-A (ACD-A) and citrate phosphate dextrose (CPD), or other activating agents, such as thrombin. Although those additive agents allow for appropriate clinical working time and manipulation, they can inhibit healing and regeneration due to the lack of biocompatibility [9,10,11,13,14,15].

The concept of low speed centrifugation is central to the preparation of the liquid form of i-PRF as well as to create a material rich in leukocytes, platelets, and growth factors, including the vascular endothelial growth factor (VEGF), transforming growth factor-beta 1 (TGF-β1), and platelet-derived growth factor (PDGF), compared to the first generation platelet-derived PRP [9,10,11,16]. According to the low speed centrifugation concept, i-PRF can be obtained by using a centrifugation speed at 60 g RCF or 700 rpm for 3 min [10,16,17]. Blood harvesting is similar to the PRF membrane method, however, the non-coating plastic tube was recommended in order to prevent the formation of early clotting in the tube. After centrifugation, whole blood is separated into three main parts based on the buffy coat layer: A yellow upper part, a buffy coat middle part, and a red blood cell containing lower part. A small syringe with an 18G hypodermic needle is used for collecting the i-PRF to be used. The harvesting method of i-PRF after centrifugation has been described as harvesting only the whole upper layer above the buffy coat [16,17], however, the amount of the upper layer harvested can vary among individuals and the position of needle tips during harvesting can also be varied based on different clinical practice. To the best of our knowledge, there is no study examining the different properties of yellow and red i-PRF.

The aim of this study was to investigate the influence of different separation techniques of i-PRF on its mechanical and biological properties. We further hypothesize that fractionation of the centrifuged plasma for i-PRF is central to the i-PRF’s physical properties and biological components. We proposed to examine two fractionation protocols producing yellow i-PRF and red i-PRF based on the fraction of centrifuged plasma above and within the buffy coat, respectively. We expected that the yellow i-PRF would have less cellular components, a denser fibrin network, and therefore have better physical properties than the red i-PRF. On the contrary, we expected that the red i-PRF would have more cellular components and therefore have better biological properties reflecting the greater release of known PRF-related growth factors, including VEGF, TGF-β1, and PDGF.

## 2. Results

**Figure 1:**
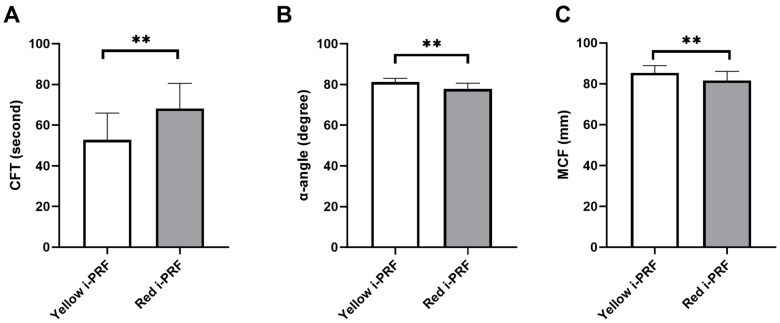
Rotational thromboelastometry (ROTEM^®^) analysis demonstrating (**A**) clot formation time (CFT), (**B**) α-angle, and (**C**) maximum clot firmness (MCF) of the yellow injectable platelet-rich fibrin (i-PRF) and the red i-PRF. ** *p* < 0.01.

Rotational thromboelastometry (ROTEM^®^, Tem International GmbH, Munich, Germany) [18] was applied to examine the viscoelastic properties of red and yellow i-PRF. The means and standard deviations for the clot formation time (CFT), α-angle, and maximum clot firmness (MCF) were 52.8 ± 13.14 s, 81.2 ± 1.81 degree, and 85.3 ± 3.56 mm, respectively, for the yellow i-PRF and 68.2 ± 12.31 s, 77.8 ± 2.82 degree, and 81.6 ± 4.50 mm, respectively, for the red i-PRF as shown in Figure 1. The results showed statistically significant differences (paired *t*-test) between the two types of i-PRF in all three properties: CFT, α-angle, and MCF, with *p*-value = 0.008, 0.004, and 0.001, respectively, as shown in Figure 1. The CFT for the yellow i-PRF was shorter than the red i-PRF. The α-angle was higher in the yellow i-PRF compared to the red i-PRF. The MCF was also higher in the yellow i-PRF compared to the red i-PRF.

**Figure 2:**
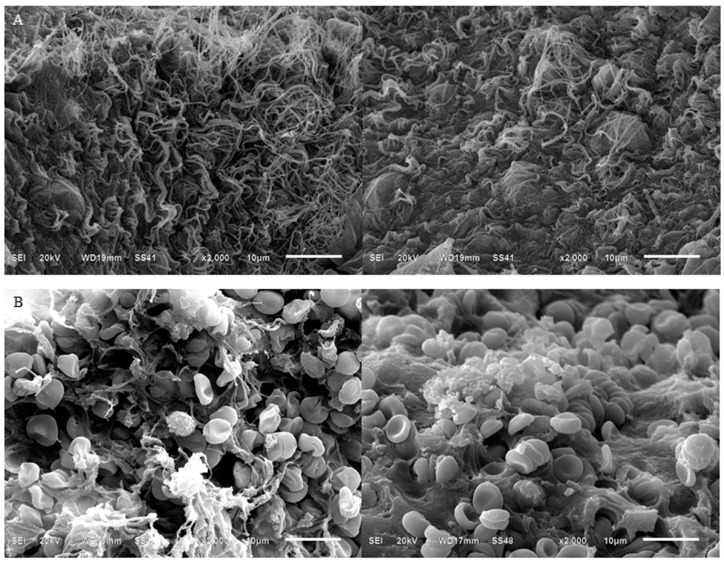
Scanning electron microscope images of i-PRF using different fraction methods. A different position of the harvesting needle led to two types of i-PRF: The yellow and red i-PRF. (**A**) The yellow i-PRF showed a dense and highly organized fibrin network. (**B**) On the other hand, the red i-PRF demonstrated more cellular components of leukocyte, platelets, and erythrocytes than the yellow i-PRF.

The examination of the i-PRF by SEM showed the fibrin network architecture and cellular components. In the yellow i-PRF, a dense fibrin network with less cellular components than the red i-PRF was observed (Figure 2). On the contrary, SEM showed that more cellular components, such as leukocyte, platelets, and erythrocytes, were in the red i-PRF compared to the yellow i-PRF. In addition, the numerous erythrocytes were enmeshed in the fibrin network. The shapes of these erythrocytes were normal, but the fibrin network appeared at a lower density and was less organized compared to the yellow i-PRF (Figure 2).

**Figure 3:**
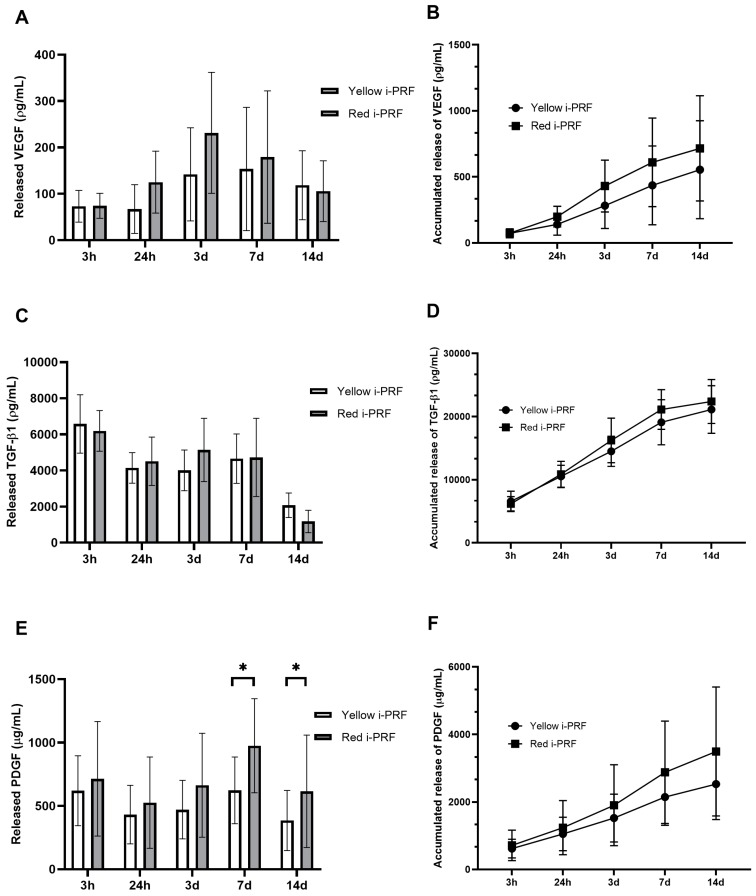
Time point releases and accumulated releases of three growth factors: (**A**,**B**) vascular endothelial growth factor (VEGF), (**C**,**D**) transforming growth factor-β1 (TGF-β1), and (**E**,**F**) platelet-derived growth factor (PDGF). * *p* < 0.05.

The growth factor concentrations were quantified at 3 h, 24 h, 72 h (3 days), 168 h (7 days), and 336 h (14 days) after preparation. There was a distinct difference between the releasing patterns for VEGF, TGF-β1, and PDGF (Figure 3). At 3 h, 24 h, and 3 days, the VEGF concentrations were increased. Three hours after preparation, the VEGF concentration of both i-PRFs was at a similar level. This level presented in the yellow i-PRF was similar at 24 h again while the red i-PRF dramatically increased. The concentrations of VEGF peaked at Day 3 for both the yellow and red i-PRF. There was a slightly higher concentration of VEGF in the red i-PRF compared to the yellow i-PRF but the difference was not statistically significant (Figure 3A). Different results were detected at Day 7 and 14 after clotting. At this time point, both i-PRFs showed a decreasing VEGF concentration. In conclusion, the released accumulation of VEGF showed a higher concentration in the red i-PRF than the yellow i-PRF (Figure 3B).

The growth factor concentration for the human TGF-β1 showed a general trend at all time points. The highest concentration was recorded at 3 h for the yellow and red i-PRF then decreased and was stable at 24 h, 3 days, and 7 days. There was a dramatic decrease of the TGF-β1 concentration for both i-PRFs at Day 14 (Figure 3C). Note that the released accumulation of TGF-β1 presented a similar trend and value for the yellow and red i-PRFs (Figure 3D). Using a paired *t*-test, there was however no statistically significant difference between the concentration of VEGF and TGF-β1 (Figure 3).

The release of PDGF was more constant throughout the experimental timeline. Analysis of the PDGF release (Figure 3E) revealed that more PDGF was released from the red i-PRF at 7 and 14 days (975.47 ± 371.24 µg/mL and 615.98 ± 443.59 µg/mL) compared with the yellow i-PRF (623.46 ± 263.30 µg/mL and 385.59 ± 237.53 µg/mL). These data were statistically significant for Day 7 (*p* = 0.02) and Day 14 (*p* = 0.03). Cumulatively, PDGF continued to release at all time points and a stronger releasing trend was observed for the red i-PRF over the yellow i-PRF (Figure 3F).

Analysis of the kinetics of the growth factors released showed that VEGF, TGF-β1, and PDGF reached peak release at Day 3, 3 h, and Day 7, respectively. On top of that, TGF-β1 was almost completely released within the first 7 days while VEGF and PDGF showed a longer releasing over 14 days.

## 3. Discussion

The main difference of i-PRF from solid PRF is the lower speed and time in centrifugation for i-PRF [10,19]. The idea of a slower centrifugation is to allow some cellular and growth factor components to stay in the final product [6,9,10,11,17,20,21,22,23,24]. The i-PRF can be used in combination with particulate bone allograft or autograft materials [25]. This advantage of a liquid formula of i-PRF allows a more efficient incorporation of the bone graft material and increases the signaling molecules throughout the whole graft.

Little information is known about the physical properties of i-PRF, which are important either when being used alone or in combination with grafting materials. This study is one of the first to apply ROTEM^®^ technology, which is commonly used in hematology research [18,26,27,28,29,30,31], to examine i-PRF. We hypothesized that compared to the lower layer—red i-PRF, the upper layer—yellow i-PRF would have superior physical properties because of its lack of cellular components. All physical properties confirmed our hypothesis. Although the yellow i-PRF was statistically superior compared to the red i-PRF in viscoelastic properties, the value of the CFT, α-angle, and MCF of both i-PRFs were similar to the reference value or slightly better compared to the standard whole blood that was measured using ROTEM^®^ [32]. In addition, the value of the CFT, α-angle, and MCF showed only a minor difference between both i-PRFs. The SEM analysis demonstrated a dense, organized, acellular fibrin network as a reason for the superior physical properties in the yellow i-PRF compared to the red i-PRF. This clinically suggests that if i-PRF is to be used mainly as filler material or enhancement stability and handling of grafting material, the yellow and red i-PRF may achieve a similar outcome.

A previous SEM analysis of leukocyte-PRF showed multi-different plasma layers that constituted of a fibrin-rich layer at the uppermost layer, followed by an enriched platelet layer, and the buffy coat layer with numerous leukocytes before the base layer of erythrocytes [33]. The SEM in our analysis demonstrated a dense, organized, acellular fibrin network as in the fibrin-rich layer at the plasma top layer of leukocyte-PRF. This characteristic might be a reason for the superior physical properties in the yellow i-PRF compared to the red i-PRF. Meanwhile, the red i-PRF showed a greater number of cells and platelets attached to the fibrin network similar to a combination of all the middle layers and the erythrocyte base layer of leukocyte-PRF together. This additional cell and platelet content in the red i-PRF might lead to better biological properties as we could observe a greater release of the growth factors.

Contrary to the physical properties, we hypothesized that the red i-PRF, which has more cellular components, would have higher release of growth factors. While there was no statistically significant difference in the releases of VEGF and TGF-β1, PDGF at later time points were significantly higher in the red i-PRF compared to the yellow i-PRF. This may be a result of the remaining platelet and cellular components shown in the SEM in the red i-PRF. Note also that the releasing level of all three growth factors was similar to previously reported levels [10]. In addition, we found that the growth factors continue to release even at Day 14. This result is similar to other PRF studies [9,10,11,17,20,21,22,23,24]. Self-cooperation of the fibrin network slowly forms the high fibrillar aggregation in PRF, which also entraps proteins and growth factors to the binding domains of fibrin molecules. In contrast, PRP is rapidly activated to gelation from a load of thrombin. Therefore, an unstable fibrin network of PRP is formed, which results in lower growth factor retention and incompatible cell homing. PRP therefore only releases growth factors in the early stage. Unlike the PRP, the kinetic release of growth factors from i-PRF is longer, up to 14 days, because of its preserved valuable components, including platelets and leukocytes. The growth factor release from this component can be sustained over a longer period of time from the cellular and acellular component trapped in a naturally progressive polymerization of the fibrin matrix [34]. On top of this, i-PRF potentially allows more growth factor attachment to the heparin-binding domain, which is the high-affinity growth factor-binding site of the fibrinogen that causes prolonged retention of the growth factor within fibrin [35]. This result suggests that when i-PRF is used in combination with particulate bone grafts, the signaling molecules for bone tissue engineering can be achieved. The red i-PRF may therefore have more benefits than the yellow i-PRF because of the greater release of the growth factor shown. In such cases, clinicians should harvest the red i-PRF just with the buffy coat.

This study demonstrated for the first time that minimal changes in the fractionation protocol for i-PRF could alter the physical and biological properties of the final product. This in turn may change the clinical outcomes/applications. These techniques need to be examined further through cell culture analysis and human clinical studies. In any case clinicians should pay particular attention to centrifugation and fractionation protocols. Deviations from the published protocols can result in variations of physical properties and biological components as seen in this study and in others [17]. In addition, further research to develop a novel biomaterial product from i-PRF is an interesting topic, where the freeze-dried or lyophilized technique may be used for sterilization [36,37].

## 4. Materials and Methods

### 4.1. Sample Collection and Preparation

**Figure 4:**
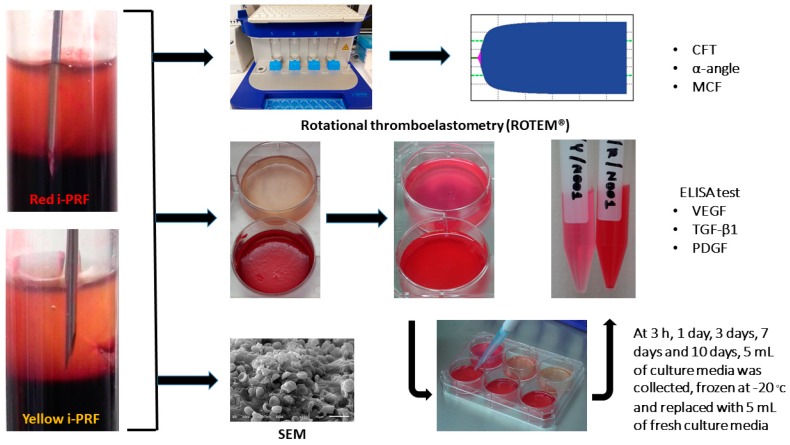
Study workflow of three experiments comparing the red and yellow i-PRF and their fractionation protocol used in this study.

The study protocol was approved by the Institutional Review Board of Human Ethic Committee of the Faculty of Dentistry, Mahidol University (COA. No. MU-DT/PY-IRB 2017/061.221). Blood samples were harvested from 10 healthy volunteers (age range 30–35, gender M/F = 2/8). The donors had no history of using antiplatelet or anticoagulant medications. All donors gave written consent for blood collection and for sample preparation and experiments (Figure 4).

Sample preparation was adopted from a previous published protocol, Miron et al. 2017 [10]. Immediately after the blood sample was collected, the sample was centrifuged at 60 g RCF for 3 min using a Duo Centrifuge (Process for PRF, Nice, France) at room temperature; 60 g RCF is equivalent to 700 rpm for this device, which has a 110 mm radius. After centrifugation, two types of i-PRF samples were harvested, yellow and red i-PRF. According to Wang et al. 2017 [24], about 1 mL of sample, either the yellow or red i-PRF, was collected. The yellow i-PRF referred to the sample harvested only in the upper liquid yellow zone above the buffy coat. The red i-PRF referred to the sample harvested from the zone, red and yellow, with the buffy coat. The bevel edge of the harvesting needle was used as a reference point (Figure 4). Subsequently, both types of i-PRF were used to observe the physical properties, including viscoelasticity and morphology, and the biological properties, which is the release of growth factors.

### 4.2. Viscoelastic Property Analysis

The viscoelastic properties of the i-PRF samples were analyzed using rotational thromboelastometry (ROTEM^®^, Tem International GmbH, Munich, Germany) [18]. ROTEM^®^ generates output from transducing changes reflecting from the viscoelastic strength of the blood sample while a constant rotational force is applied. Three parameters were analyzed and digitally recorded: Clot formation time (CFT), α-angle, and maximum clot firmness (MCF). The CFT represents the speed at which a solid clot forms, which is primarily influenced by the platelet function, fibrinogen, and coagulation factors. The CFT measures a duration from the clot initiation until it reaches the 20 mm amplitude. The α-angle is a measure to observe the dynamics of clot formation, which represents the acceleration of the fibrin network formation and the amount of cross-linking build up. The MCF is a measure for stability of the clot following the polymerization process.

### 4.3. Scanning Electron Microscopy (SEM)

The qualitative analysis of the morphological changes of the yellow and red i-PRF was performed using SEM (JEOL, Peabody, MA, USA). The SEM examination was used to observe blood elements present in the fibrin extracellular matrix. Cellular components such as leukocytes, platelets, and erythrocytes were examined and recorded. The density of the fibrin network was also analyzed. The i-PRF samples immediately after completing their clot formation were fixed in 2% glutaraldehyde in Dulbecco’s phosphate buffered saline (DPBS) buffer with a pH of 7.4 overnight. Next, samples were dried in the desiccator, then sputtered with 20 nm gold. SEM photography was then performed at 5 to 10 kV using 100× to 2000× magnifications.

### 4.4. Enzyme-Linked Immunosorbent Assay (ELISA)

ELISA kits were used to quantify the released growth factors: VEGF, TGF-β1, and PDGF (R&D systems Minnesota, United States). Approximately 9 mL of collected i-PRF from each protocol was transferred into a 6-well plate. The plate was then placed in a 5% CO_2_ incubator at 37 °C for 30 min to allow for complete clotting formation. Afterwards, 5 mL of Minimum Essential Medium Eagle-Alpha Modification (α-MEM) with nucleosides (Gibco, USA) was added to each sample. The samples were further incubated at 37 °C to allow for the release of growth factors during a 3 h to 14 days period of study. At 3 h, 24 h, 72 h (3 days), 168 h (7 days), and 336 h (14 days), 5 mL of culture media was collected, frozen at −20 °C, and replaced with 5 mL of additional fresh culture media. The concentrations of VEGF, TGF-β1, and PDGF were measured according to the manufacturer’s instructions. Optical density was assessed using a microplate reader at 450 nm. The measurements were performed in triplicates.

### 4.5. Statistical Analysis

Statistical assessment was performed using SPSS version 17.0. All data were presented as the mean value ± standard deviation (SD). The data were tested for the distribution by using a Kolmogorov-Smirnov test and Levene’s test to confirm the normality and equal variance assumptions of the data. The data, assuming normal distribution, were then analyzed using paired *t*-tests to compare the differences between two groups in each experiment. The level of significance used was *p* < 0.05.

## 5. Conclusions

This study suggests that fractionation protocols in i-PRF preparation can alter the final product’s physical and biological properties. Clinicians should pay attention to the applications of i-PRF in combinational use with other grafting materials. For the handling and stability of grafting material application, the yellow and red may provide the final i-PRF product with a similar outcome. For the use of i-PRF in enhancing biological properties, the red i-PRF collected from the sample with the buffy coat, which released a higher level of growth factors, in particular, PDGF, should be used.
